# Minutes of the Convention Assembled in the City of New-York, on the 18th Day of August, 1840, for the Purpose of Forming a National Dentists' Society, Together with an Account of the Organization of Said Society, with Its Constitution and By-Laws

**Published:** 1840

**Authors:** Solyman Brown

**Affiliations:** Secretary.


### NATIONAL DENTISTS' SOCIETY.

---

*Extracts from the Minutes of a* CONVENTION OF DENTISTS, *assembled at the American Hotel, in the City of New-York, on the 18th day of August, in the year of Our Lord, one thousand eight hundred and forty.*

At ten o'clock, A. M., on motion of HORACE H. HAYDEN M. D. of *Baltimore,* the convention was called to order by nominating ELISHA BAKER, of the *City of New-York* as chairman, and SOLYMAN BROWN, A. M. of the same city, Secretary.

Present personally :—Horace H. Hayden, M. D. of Baltimore ; L. S. Parmly, of New Orleans ; Elisha Baker, of New-York ; Patrick Houston, M. D. of Charlston, S. C ; Eleazar Parmly, M. D. of New-York ; Enoch Noyes, of Baltimore ; Vernon Cuyler, M. D. of Hartford Conn ; John Lovejoy of New-York ; Chapin A. Harris, M. D, of Baltimore ; Jahial Parmly, of New-York ; Joseph N. Foster, M. D. of New-York ; Augustus Woodruff Brown, of New-York ; Charles O Baker, of New-York ; J. Smith Dodge of New-York ; and Solyman Brown, of N. Y.

Present by proxy :—Lewis Roper, M. D. of Philadelphia ; Daniel Harrington, of Philadelphia. Present by letter :—J. McIlhany, of Philadelphia ; E. B. Gardette of Philadelphia; Edward Hudson, M. D. of Philadelphia; William Arnold, M. D. of New-York ; Edward Maynard, M. D. of Washington City; Jas. S. Gunnell M. D. of Washington City ; Isaac J. Greenwood, of New-York ; Josiah F. Flagg, M. D. Boston, and S. Spooner, of New-York. Present by invitation :—Leonard Macall, M. D. of Batimore, John Trenor, M. D. of New-York ; Elihu Blake, of New-York ; Dr. Greenwood, of Boston ; Dr. Keep, of Boston ; Daniel Harwood, M. D. of Boston ; Samuel W. Parmly, of New-York ; E. G. Tucker, M. D. New-York ; R. Burr, of Philadelphia ; F. B. Chewning, Richmond Va.; S. M. Sheppard, Petersburg Va. Joshua Tucker, M. D. Boston.

On motion of Dr. Harris, Resolved that it is the opinion of this covention that the science of Dental Surgery would be advanced, and the interests of all well informed practitioners and community at large, promoted, by the formation of a national society of dentists; therefore resolved furthermore, that in order to the accomplishment of this object, a committee consisting of three persons, be appointed by the chairman of this convention, to prepare the form of a constitution for such a society with instructions to report the same as soon as they shall have performed their duty.

The foregoing resolutions having passed unanimously, Dr. Harris, Mr. S. Brown, and Dr. Cuyler, were appointed on said committee.

For the purpose of giving said committee time to prepare their report, and for the greater convenience of the convention, it was, on motion, resolved that the meeting adjourn to the residence of the Messrs S. & A. W. Brown No. 13 Park Place, and to re-assemble at 12 o'clock this day.

Tuesday Aug. 18th 12 o'clock, M.—The Convention re-opened at 13 Park Place—Present as before.

The chairman of the committee on the constitution made the following report. That they had complied with the instruction of the convention, and with the consent of the chairman would present to the secretary the form of a constitution for the National Society of Dental Surgeons.

Whereupon Dr. Hayden expressed his desire of making a few brief remarks before submitting the constitution to the consideration of the meeting.

The formaton of a National Society of Dentists, he remarked, had been long a favorite project with himself; and he was happy to say, that he had never found himself wholly alone in this desire. Many years ago he had consulted with the elder Hudson of Philadelphia, who was favourably inclined to such an association. On a visit to Boston and other Eastern cities he had consulted his professional brethern on the same question. He found them ready to co-operate in any practicable scheme for the elevation of the profession, but they were inclined to doubt whether any thing useful could be accomplished by any other than individual efforts. Of any advantages that could result from associated influence, they were mostly skeptical, especially in Boston. It was somewhat otherwise with a few of the profession in the city of New-York. Here several of his brethern were anxious to disseminate knowledge, cultivate social intercourse, and make an effort to elevate the profession of Dental Surgery from its present neglected condition to the dignity of a science. In Philadelphia, Baltimore, Washington and a few other cities his plan was received with favor by some of the individuals with whom he consulted.

The speaker adverted in the next place to the condition of the dental art at the time he entered upon its duties forty three-years ago. Then, said he, the name of dentist was a reproach and a by-word; but he resolved that his son whom he intended to educate to his own calling, should find the profession so far improved and exalted as that it should be no longer a disgrace to be called a dentist. To this end he enquired of the elder

Greenwood of New-York for books on the subject of dental theory and practice. That gentleman informed him of the work of John Hunter which he procured. Soon after he obtained possession of some few other works both in French and English. From that time he has seen the science assuming more and more importance in the public eye, while men of leaıning, worth and genius have been added from year to year to the rank of its professors.

At the present period, continued the speaker, renewed efforts are making in England, France and Scotland, to place our profession on still higher ground than it has yet attained ; and shall we of these United States of America, remain inactive in this grand endeavour ? For myself, said he, I cannot brook defeat in this favorite undertaking. I prefer rather to make renewed and redoubled efforts to secure success. If, like the Jews, we are scattered and despised, may we not like that persecuted people, at least hope for a day of restoration to our promised land! There are indeed many obstacles still to overcome. A new race of Canaanites must be expelled from our borders. Many errors must be exploded, and much ignorance must be dispelled, before the light of truth will beam clearly upon our path; but with diligence, zeal and perseverance, we are certain of ultimate success. Let us, therefore, go forward in the good cause, unintimidated by the skepticism of the faithless, the fears of the timid, or the apathy of the selfish. If there are some who prefer to plod onward in the path of private enterprise, let us unite our efforts in one great social endeavor to elevate our profession from the degraded condition to which it has sunk, and in which it must ever remain until the high minded and well educated amongst its practitioners shall unitedly arise and shake themselves from the dust.

Dr. Hayden then offered a resolution that the Secretary proceed to the reading of the Constitution, as reported by the Committee appointed for that purpose ; whereupon the Secretary, by order of the Chairman, read as follows :—

### PREAMBLE

### OF THE OBJECTS OF THE SOCIETY.

### ARTICLE I.

The objects of this Society are to promote union and harmony among all respectable and well informed Dental Surgeons ; to advance the science by free communication and interchange of sentiments, either written or verbal, between members of the Society, both in this and other countries ; in fine, to give character and respectability to the profession, by establishing a line of distinction between the truly meritorious and skilful, and such as riot in the ill gotten fruit of unblushing impudence and empiricism.

### ART. II. *Of the name of the Society.*

The Society shall be known and designated by the name and title of "The American Society of Dental Surgeons."

ART. III. *Of the Officers of the Society.*

Sec. 1. The Officers of the Society shall consist of a President, and three Vice Presidents, a recording and a corresponding Secretary, a Treasurer, a Librarian, an Executive, an Examining and a Publishing Committee.

Sec. 2. The election of the above named Officers shall be by ballot, at a regular annual meeting of the Society—a majority of votes determining the election.

ART. IV. *Of Members of the Society.*

There shall be two classes of members known and recognized as acting Members or Fellows, and Honorary Members; the former consisting of those who subscribe this constitution either personally or by proxy, and pay into the treasury the annual dues required by this constitution; and the latter embracing such members as are merely elected to membership.

ART. V. *Of the Requisitions of Membership.*

Sec. 1. Each and every Acting Member of this society shall either have been such by virtue of his attendance in person, by proxy, or by letter, at the time of its formation, or as shall be afterwards elected as prescribed by this constitution, and subscribe to the same.

Sec. 2. Each and every Acting Member shall pay into the Treasury of the Society the annual sum of Five Dollars, for the benefit of its funds.

Sec. 3. Each and every Acting Member shall be required to attend the Society at least once in three years, unless excused by the Society.

ART. VI. *Of the Election of Members.*

Sec. 1. All members of the Society, excepting those who were Acting Members at the time of forming the same and establishing this constitution, shall be elected as follows, viz.—The candidate for membership shall be proposed at a regular meeting by the Executive Committee, whereupon two Tellers shall be appointed by the presiding Officer, who shall collect the ballots, consisting of slips of paper having plainly written on them either *Yea* or *Nay*. In case two thirds of the members present shall vote in the affirmative the candidate shall be declared to have been duly elected a member of this society.

ART. VII. *Of the expulsion of Members.*

Sec. 1. Any member of the society may be expelled for immoral conduct, malpractice in business or other sufficient cause, on motion of one member, seconded by another at any regular meeting of the society, in which case a majority of three-fourths of the members present, shall be required.

ART. VIII. *Of the Meetings of the Society.*

Sec. 1. The Meetings of the Society shall be held annually by adjournment, from time to time, and from place to place, agreeably to the will of the Society.

ART. IX. *Of the resources of the Society.*

Sec. 1. The Society may receive contributions of money, books, or other property, which may be either used or sold in aid of its purposes.

Sec. 2. Each and every candidate examined and admitted to membership, shall pay to the Treasurer a fee of twenty-five dollars, before receiving his diploma.

Sec. 3. Each and every person who shall become an active member of the Society by election, shall pay ten dollars for the benefit of its funds, which shall entitle him to a diploma.

ART. X. *Of the disposition of the funds of the Society.*

Sec. 1. The funds of the Society may be appropriated to the purchase of lands, tenements, chemical or philosophical apparatus,—the publishing of books, tracts, and other papers and documents ; and to such charitable objects as shall hereinafter be enumerated.

ART. XI. *Of the distribution of the effects of this Society, in the event of its final dissolution.*

Sec. 1. In case a dissolution of the Society shall at any time be proposed, a meeting shall be called specially for that purpose ; and the consent of three-fourths of its members be required to effect it.

Sec. 2. Should the Society thus be dissolved by its own act, the property belonging to it shall be sold by order of the President, or by any three Fellows who shall obtain authority from a majority of the living and subscribing members, and the assets of sale shall be equally divided among the subscribing members.

ART. XII. *Of the indispensible qualifications of candidates who have never yet entered upon professional practice.*

Sec. 1. The candidate shall be at least twenty one years of age ; shall have a good English education ; shall produce evidence of unexceptionable moral character ; and shall have studied and practiced for the full term of two years with some practical dentist known as such to this Society.

Sec. 2. No candidate for membership, who has the diploma of any Dental College, regularly chartered in any of the United States, shall be subjected to re-examination by the Examining Committee of this Society ; but shall be entitled to a Diploma by complying with the By-Laws.

ART. XIII. *Of the Quorum required to transact business; and of the contingency of there being no quorum present at any meeting.*

Sec. 1. Seven acting Members, or Fellows, of the Society, besides the President or Chairman, shall be necessary for the transaction of business, at the opening of any annual meeting.

Sec. 2. In the event that a quorum cannot be obtained at any regular meeting of the Society, the time of meeting on the following year shall be the second Tuesday of August, and the place the same as on the preceding year.

ART. XIV. *Of the charitable objects of the Society.*

Sec. 1. Any surplus monies in the treasury, may be appropriated, at any regular meeting of the Society, for the aid and relief of the widows and orphans of deceased members; or for the benefit of living members reduced to want, by sickness or other calamity; it being provided that no such appropriation shall be made without the consent of two-thirds of the members present.

Sec. 2. Other charitable or patriotic appropriations may be made at any regular meeting of the Society, by a majority of three-fourths of the members present.

Sec. 3. The President may at any time, give pecuniary assistance to any member, or to the surviving family of any deceased member, out of any monies in the treasury, deposited there by benevolent individuals.

ART. XV. *Of alterations and amendments of this Constitution.*

Sec. 1. Any alteration, amendment, or revision of this Constitution, may be made at any regular meeting of the Society, by the joint assent of three fourths of the Fellows present at such meeting.

HORACE H. HAYDEN.
ELEAZAR PARMLY.
VERNON CUYLER.
ELISHA BAKER.
JOHN LOVEJOY.
L. S. PARMLY.
CHAPIN A. HARRIS.
CHARLES O. BAKER.
P. HOUSTON.
ENOCH NOYES.
JAHIAL PARMLY.
SOLYMAN BROWN.
J. SMITH DODGE.
JOSEPH H. FOSTER.
A. WOODRUFF BROWN.

The foregoing Constitution having been read, article by article, and amended, was unanimously adopted; whereupon Eleazar Parmly, after stating that the necessity of leaving the city on indispensable business, would deprive him of the pleasure of taking further part in the proceedings of the Convention, read the following communication, which was ordered to be deposited in the archives of the society:

*Mr. Chairman,*

I feel myself happy in being of the number of those whose names shall be transmitted, in the act which we have just performed, to the latest posterity; and whose memories will live in the history of our profession, long after our mortal remains shall have been gathered to the home of our fathers. We can easily imagine the glow of patriotic enthusiasm which animates, at this day, the hearts of those who can trace the blood which flows in their veins to the heroes of the American Revolution, who signed their names to that great political document which secures to us our national independence. And why is it, that this exulting self-congratulation and honorable pride, are felt by the descendants of that band of patriots whose memories shall live in the hearts of their countrymen as long as they remain the happy citizens of this glorious and independent republic? It is in consequence of the struggles through which they passed in order to acquire the power of calling themselves a free and sovereign people; and of the sacrifices which they made for the good of their common country.

Will not a similar feeling kindle in the breasts of those who shall come after us, when a small pamphlet shall fall into their hands, either by accident or design, bearing the names of those individuals of this assembly, to whom they can claim the affinity of kindred blood; may not a noble pride be felt by our children, and even by our children's children, when they read that their ancestors were of the little band who met from distant cities, to rescue a noble Science from the degradation to which it had been reduced by ignorance, selfishness and quackery! And will it be no source of pleasing recollection to ourselves in the subsequent stages of our existence, that on such a day of such a year, we met each other in this place, and signed a document which declared ourselves members of a profession which afforded ample scope for all the moral, intellectual and physical energies of our nature engaged for the general good of our fellow men!

Our forefathers met with opposition and oppression in every attempt to establish their rights as men. We too have met in former years with indignities and sarcasms in every attempt to sustain our privileges as members of our profession. We have, I presume, all of us, the mortification to know, that in this country, more particularly than in any other, the community at large have been accustomed to regard men and gentlemen too, as unfit for the drawing rooms of the rich and the fashionable, if they attach themselves to the profession which we are resolved to honor and to elevate. It is time to blot from the annals of society this degrading feature, and to claim for ourselves a full equality with the honorable avocations of our fellow men. We owe it to ourselves; and we owe it especially to our children.

The support of the Constitution which has just been adopted, is, in my opinion, an object worthy of our vigorous and united efforts. Personal fame and aggrandizement, should never be allowed to constitute predominant ingredients in the composition of our professional ambition.

Nobler motives shall animate our zeal, while engaged in laying the corner stone of a structure which, completed by our successors, shall, at some future day, command the respect, if not the admiration, of the world.

Without such an object, our present undertaking would be unworthy of men ; and our present meeting a school boy amusement. But I trust we have come together from distant cities of the union, with a settled determination to carry out in detail, with manly vigor, the objects proposed by that instrument which we have just declared to be a fit constitution for an American Society of Surgeon Dentists.

It will be to each of us a fresh stimulus to exertion, that the failure of our plans is not only confidently predicted, but ardently desired by many whose professional ambition knows no impulse beyond a thirst for gold. Besides, should we fail in our object of giving character and consistency to the profession ; of imparting to it the form of a regular Science ; and of uniting its members for the common good,—we shall discourage, perhaps, for years to come, a similar attempt. On the other hand, if by persevering diligence, we not only deserve, but command success ; if our proposed Society should effect the object we have in view ; and if the prophets among our enemies, like those of Baal, should invoke in vain the interposition of their gods ; then, indeed, shall we have performed a work worthy of the high ambition of christians and of men ; and the children who come after us, shall not find themselves degraded by the calling of their progenitors.

With these sentiments thus hastily thrown together, I must bid you adieu, after expressing my hearty co-operation in all the plans of usefulness which your wisdom may devise. ELEAZAR PARMLY.

August 19, morning Session. The Convention assembled agreeably to adjournment, at the residence of Dr. BAKER, No. 6 Warren Street. After the reading and approval of the minutes of the preceding session ; and after the reading of the Constitution as transcribed by the Secretary, which was also approved ; it was, on motion, *resolved,* That a Committee on By-Laws be appointed from the Chair, whereupon, Messrs. Harris, S. Brown and Noyes, were named on that Committee.

The following are the By-Laws reported by that committee, and ultimately amended and approved by the Convention.

—

### BY-LAWS.

### ART. I. *Of the duties of Officers.*

SEC. 1. It shall be the duty of the President to preside at all Meetings of the Society, when present ; to sign the diplomas conferred on members during his term of office ; to confer all honorary distinctions awarded by the Society, whether degrees of Dental Surgery, medals or other testimonials ; and to draw upon the treasurer for all monies appropriated by the Society.

SEC. 2. It shall be the duty of the first Vice President to preside at all meetings of the Society in the absence of the President ; and to perform the other prescribed duties of the President, in case of his death, resignation, sickness, or other disability.

Sec. 3. It shall be the duty of the second and third Vice Presidents, to perform the duties of the above named officers, during their absence, or inability.

Sec. 4. A President *pro-tem.* shall be appointed to preside at any meeting, when the President and Vice Presidents are all absent.

Sec. 5. It shall be the duty of the Recording Secretary, to note in a book kept for the purpose, all the proceedings of the Society, during the term of his office, and to furnish copies of any parts thereof, at the order of the President, or his substitutes; and, also, to deliver the said book and proceedings to his successor in office. He shall also keep the seal of the Society, together with the copper-plate, from which diplomas, certificates, or degrees, are struck; also all the records belonging to the Society, designed to be preserved; and he shall furthermore countersign all diplomas, and set the seal of the Society to all instruments requiring the same; he shall also notify every member of the time and place of meeting of the Society, at least two months before the time of said meeting.

Sec. 6. It shall be the duty of the Corresponding Secretary, to conduct the domestic and foreign correspondence of the Society, in accordance with the advice of the President, or the express will of the Society communicated to him by the recording Secretary, and at his own discretion, in cases of emergency, provided it does not interfere with any of the prescribed rules of the Society.

Sec. 7. It shall be the duty of the Treasurer to keep all the monies of the Society committed to his trust; to pay them over to the order of the President, countersigned by the Secretary, and to keep a correct account of the same in a book or books kept for that purpose.

Sec. 8. It shall be the duty of the Librarian to keep the books belonging to the Society's Library; to obtain insurance on the same; and to take receipts of all persons who receive books from his hands by order of the Society; and in case of failure of the member receiving such book, to return it in twelve months, he shall forfeit, and pay to the Society, four times its price.

Sec. 9. It shall be the duty of the Executive Committee, to examine the accounts of the Treasurer, and report the same to the Society at its annual meetings; to see that all the rules and regulations of the Society are duly enforced and executed; and it shall also be the duty of such committee, to enquire into the professional reputation and character of all candidates proposed, or who may apply for membership, and report the same to the Society, which, being satisfactory, such candidates shall be ballotted for, for admission into the same.

Sec. 10. It shall be the duty of the Examining Committee, to appoint a time and place, at least once in each year, for the examination of applicants for the degree of Doctor of Dental Surgery; to examine such persons strictly and faithfully, in relation to their qualifications to enter upon the practice of the profession; and their certificate shall entitle the holder to receive the diploma of the Society conferring the degree of Doctor of Dental Surgery.

SEC. 11. It shall be the duty of the Publishing Committee, to superintend the printing and publishing of all books, tracts, and other documents, issued by the Society.

ART. II. *Of the Emoluments of Office.*

SEC. 1. No officer shall receive any emolument unless in cases of special appropriation, excepting the Recording Secretary, whose duty it shall be to attend the meeting of the society next subsequent to his election; and who shall be entitled to his travelling expences and the sum of five dollars per diem during his necessary absence from business, in all cases where he is compelled to leave his place of residence.

ART. III. *Of the powers vested in the Vice Presidents.*

SEC. 1. Each of the Vice Presidents may, if there be a sufficient number of the members of this society residing in his immediate vicinity, convene them at any time, and when so convened, they shall have power to elect such officers from among their own number as may be necessary for the transaction of whatever business of a local nature may come before them; and it shall be obligatory on them to report their proceedings regularly at each annual meeting of the parent society.

ART. IV. *Of the Privileges of Members.*

SEC. 1. Each and every acting Member, or Fellow of the Society, shall be entitled to debate and vote on all questions agitated at any meeting thereof, agreeably to the By Laws and Standing Rules and Regulations of the Society; and he shall moreover be eligible to any office in the gift of the Society; and, furthermore, he shall be entitled to a Diploma, or Degree of Doctor of Dental Surgery.

SEC. 2. Each and every Honorary Member shall be entitled to a seat at all meetings of the society;—shall have the privilege of debating all questions not involving pecuniary expenditure; and shall receive the Diploma, or Degree of Doctor of Dental Surgery by paying therefor to the Treasurer of the Society, the sum of Ten Dollars.

ART. V. *Of the Appointment of Members to certain duties.*

SEC. 1. It shall be the business of the Society, at each annual meeting, to appoint five of its Members to prepare Dissertations on some subjects connected with the profession; and also one Member to deliver an opening Address at the subsequent meeting.

SEC. 2. It shall moreover be the duty of the Society, from time to time, to appoint certain individuals to prepare Essays or any other Documents ordered by the Society.

ART. VI. *Of Awarding Premiums.*

SEC. 1. This Society may award premiums to Members, for dissertations on subjects that may at any time be specified by the Society at an annual meeting; also, for important improvements in Mechanical Dentistry, and the manufacture of Incorruptible Teeth; and also for any other suitable object.

Sec. 2. The question of preference in all such cases, shall be determined by the judgment of a Special Committee, appointed by the Society.

Sec. 3. Unsuccessful dissertations shall be at the disposal of their respective authors.

ART. VII.

Sec. 1. No Member shall speak more than twice to any question, without express permission of the Society, and then only fifteen minutes at each time.

Sec. 2. Any member shall be liable to be called to order for personal recrimination of any other member; or for wandering in his remarks from the subject of debate.

ART. VIII.

These By-Laws may be altered or amended, at any annual meeting of this Society, by a vote of two thirds of the members present.

Wednesday, August 19 — Evening Session. The Convention met at the house of Dr. Baker, agreeably to adjournment, whereupon, on motion to that effect, the Convention resolved itself into the American Society of Dental Surgeons.

On motion, *resolved,* That all the individuals, who have attended the Convention in person, by letter or by proxy, as well as those who have been invited to attend the same, be considered as members of this Society.

After adopting the Constitution and By-Laws, as recommended by the Convention, the Society proceeded to the election of its officers for the ensuing year, when the following gentlemen were chosen to the respective offices agreeably to the provisions of the Constitution and By-Laws of the Society.

HORACE H. HAYDEN, M. D., *President.*

JOSIAH F. FLAGG, M. D., *1st Vice President.*

ELEAZAR PARMLY, M. D., *2nd Vice President.*

E. B. GARDETTE, *3d Vice President.*

SOLYMAN BROWN, A. M., *Recording Secretary.*

CHAPIN A. HARRIS, M. D., *Corresponding Sec.*

ELISHA BAKER, *Treasurer.*

J. H. FOSTER, M. D., *Librarian.*

On motion, *resolved*, That the three Committees required by the Constitution, consist of five members each, who were duly elected as follows:

| | |
|---|---|
| JAHIAL PARMLY,<br>LEVI S. PARMLY,<br>ENOCH NOYES,<br>LEWIS ROPER,<br>NATHAN C. KEEP, | EXECUTIVE COMMITTEE. |
| J. SMITH DODGE,<br>DANIEL HARWOOD,<br>DANIEL HARRINGTON,<br>LEONARD MACALL,<br>P. HOUSTON, | EXAMINING COMMITTEE. |
| SAMUEL W. PARMLY,<br>S. SPOONER,<br>JOHN LOVEJOY,<br>EDWARD HUDSON,<br>JAMES S. GUNNELL, | PUBLISHING COMMITTEE. |

On motion the Society proceeded to the election of Members in accordance with the provisions of the Constitution and By Laws, when the following persons were duly elected as Members of the Society.

| | |
|---|---|
| John Harris, M.D. *Georgetown, Ky.* | Doct. Townsend, *Philadelphia.* |
| Cha's Hayden, *Washington City.* | H. M. Hayden, *Baltimore.* |
| F. H. Clark, *Baltimore.* | Oliver Holmes, *Baltimore.* |
| B. Monafeldt, *Charleston, S. C.* | Ludolph Parmly, *Mobile.* |
| Doct. Fisher, *Providence, R. I.* | Nicholas Clute, *Louisville, Ky.* |
| Doct. Rogers, *Cincinnatti.* | Elbridge Bacon, M. D., *Boston.* |
| R. Somerby, *Louisville, Ky.* | J. D. McCabe, *Richmond, Va.* |
| Samuel Avery, *N. Orleans.* | B. A. Rodriguez, *Charleston, S. C.* |
| Doct. Sumner, *Providence.* | Jahial Parmly, *Savannah.* |
| W. B. Scott, *Raleigh, N. C.* | A. B. Hayden, *Savannah.* |
| J. A. Cleveland, *Charleston, S. C.* | Doct. Straw, *Bangor, Me.* |
| O. P. Laird, *Columbus, Ga.* | M. K. Bridges, *Brooklyn, N. Y.* |

On motion, *resolved*, That the Society approve of the Establishment of the Baltimore College of Dental Surgery, and will co-operate with the other friends of that institution in promoting its designs.

Thursday morning, August 20. An election was held to appoint a member to deliver an address at the opening of the next meeting of the Society—when Dr. Hayden, the President, was chosen for that purpose.

On motion, *resolved*, That five persons be chosen to deliver dissertations on subjects connected with Dental Theory, or Practice, at the next annual Meeting of the Society; whereupon, Solyman Brown, J. Smith Dodge, Chapin A. Harris, J. H. Foster, and Elisha Baker, were duly appointed.

*Resolved*, That the Society proceed to appoint twelve members to prepare Essays, on specified subjects, for the benefit of the profession. The following gentlemen were appointed.

1. L S. Parmly, on the best method of saving the natural teeth.
2. E. Baker, on ulcerated fangs, and the methods of cure.
3. E. Parmly, on the necessity of stopping carious teeth.
4. J. F. Flagg, on the Ligamentum Dentis, so called.
5. C. A. Harris, on the necessity of artificial teeth.
6. S. Brown, on the necessity of regulating the natural teeth.
7. J. Smith Dodge, on the extraction of diseased teeth.
8. L. Mackall, on Diseases of the Gums.
9. E. Noyes, on deciduous teeth.
10. J. S. Gunnell, on salivary Calculus.
11. John Harris, on the propriety of filing the teeth.
12. V. Cuyler on tooth ache.

Thursday evening, August 20th. Society met agreeably to adjournment, at the residence of Messrs. Tucker & Foster, No. 3 Park Place.

On motion, the meeting proceeded to the election of Honorary Members, in addition to those already chosen, whereupon the following new members were added to the Society.

Samuel Cartwright, of *London.*
Robert Nasmyth, of *Edinburg.*
Alexander Nasmyth, of *London.*
C. S. Brewster, of *Paris.*
John T. Edmonds, of *London.*
James McPherson, *Glasgow, Sc.*
A. G. Becht, *Hague, Netherlands.*
H. N. Fenn, *Rochester, N. Y.*
James Taylor, *Crawfordville, Ia.*
S. Blanding, M.D. *Columbia, S. C.*
Leonard Koecker, of *London.*
Thomas Bell, of *London.*
C. F. Delabarre, of *Paris.*
David Wemyss Jobson, of *Edinburg.*
E. Gidney, *Manchester, Eng.*
Edward Taylor, *Maysville, Ky.*
Solomon Keep, *Boston.*
Mons. Le Maire, *Paris.*

On motion—*Resolved*:—That a Committee on Seals, Diplomas, &c. be appointed. Dr. Harris, Dr. Maynard and Dr. Macall, were named by the President.

On motion—*Resolved*:—That the Corresponding Secretary notify the Members elect, of their having been chosen Members of the Society, and furnish each a copy of the Constitution and By Laws.

On motion—*Resolved*:—That Eleazar Parmly, M. D., be appointed as the Agent of the Society, to present a petition to the Legislature of the State of New-York, asking for a Charter, with power of conferring the degree of Doctor of Dental Surgery.

On motion—*Resolved* :—That this Society approve of the establishment and management of the American Journal of Dental Science, and will aid its distribution and design.

On motion—*Resolved*:—That the Society now adjourn to the second Tuesday of August, A. D. 1841, to meet at the United States Hotel, in the City of Philadelphia, at ten o'clock in the morning of that day.

SOLYMAN BROWN,

*Secretary*.

