# Correction

**Published:** 1841

**Authors:** 


*Page* 158, in place of what is said in two last paragraphs, and to the word, on 159th page, 3d line, procured, read the following :

The formation of a National Society of Dentists,he remarked, had been long a favourite project with himself ; and he was happy to say, that he had never found himself wholly alone in this desire. As long since as the year 1817, he had consulted with the late Doctor Edward Hudson, of Philadelphia, who was favourably inclined to such an association.—Other gentlemen of the profession, however, were not inclined to make an effort in the business, although gentlemen of the Medical Faculty, (to their honor be it spoken) uniformly approved of the plan.

The prospect of succeeding, however, appearing rather unfavourable, he had mostly relinquished the plan until finding that his son manifested a desire to pursue the same profession, and contemplating a tour to the North in 1829, he resolved to make another effort amongst his professional friends and acquaintances, and in this instance, he met with much more encouragement, but not however, sufficient to justify a belief that the plan would succeed until the year 1838, when, in a tour to the north, he made the last attempt.

The speaker adverted in the next place, to the condition of the dental Art, at the time when he entered upon its duties, forty-three years ago. Then, said he, the name of a dentist was a reproach and a by-word. But viewing it as a subject susceptible of great and beneficial improvements, by well written essays or works, and not knowing at the time whether any such had ever been published, he applied to the elder Mr. Greenwood, of New-York, (then dentist to General Washington) who informed him, that he knew but one work that had ever been published, and that was John Hunter's on the Natural History of the Teeth, which he procured.

